# A Primer on Metagenomics

**DOI:** 10.1371/journal.pcbi.1000667

**Published:** 2010-02-26

**Authors:** John C. Wooley, Adam Godzik, Iddo Friedberg

**Affiliations:** 1 Community Cyberinfrastructure for Marine Microbial Ecology Research and Analysis, California Institute for Telecommunications and Information Technology, University of California San Diego, La Jolla, California, United States of America; 2 Program in Bioinformatics and Systems Biology, Burnham Institute for Medical Research, La Jolla, California, United States of America; 3 Department of Microbiology, Miami University, Oxford, Ohio, United States of America; 4 Department of Computer Science and Software Engineering, Miami University, Oxford, Ohio, United States of America; University of California San Diego, United States of America

## Abstract

Metagenomics is a discipline that enables the genomic study of uncultured microorganisms. Faster, cheaper sequencing technologies and the ability to sequence uncultured microbes sampled directly from their habitats are expanding and transforming our view of the microbial world. Distilling meaningful information from the millions of new genomic sequences presents a serious challenge to bioinformaticians. In cultured microbes, the genomic data come from a single clone, making sequence assembly and annotation tractable. In metagenomics, the data come from heterogeneous microbial communities, sometimes containing more than 10,000 species, with the sequence data being noisy and partial. From sampling, to assembly, to gene calling and function prediction, bioinformatics faces new demands in interpreting voluminous, noisy, and often partial sequence data. Although metagenomics is a relative newcomer to science, the past few years have seen an explosion in computational methods applied to metagenomic-based research. It is therefore not within the scope of this article to provide an exhaustive review. Rather, we provide here a concise yet comprehensive introduction to the current computational requirements presented by metagenomics, and review the recent progress made. We also note whether there is software that implements any of the methods presented here, and briefly review its utility. Nevertheless, it would be useful if readers of this article would avail themselves of the comment section provided by this journal, and relate their own experiences. Finally, the last section of this article provides a few representative studies illustrating different facets of recent scientific discoveries made using metagenomics.

## Introduction

For most of its history, life on Earth consisted solely of microscopic life forms, and microbial life still dominates Earth in many aspects. The estimated 5×10^30^ prokaryotic cells inhabiting our planet sequester some 350–550 Petagrams (1 Pg = 10^15^ g) of carbon, 85–130 Pg of nitrogen, and 9–14 Pg of phosphorous making them the largest reservoir of those nutrients on Earth [1]. Bacteria and archaea live in all environments capable of sustaining other life and in many cases are the sole inhabitants of extreme environments: from deep sea vents with temperatures of 340°C to rocks found in boreholes 6 km beneath the Earth's surface. Bacteria, archea, and microeukaryotes dominate Earth's habitats, compound recycling, nutrient sequestration, and, according to some estimates, biomass. Microbes are not only ubiquitous, they are essential to all life, as they are the primary source for nutrients, and the primary recyclers of dead matter back to available organic form. Along with all other animals and plants, the human condition is profoundly affected by microbes, from the scourges of human, farm animal, and crop pandemics, to the benefits in agriculture, food industry, and medicine to name a few. We humans have more bacterial cells (10^14^) inhabiting our body than our own cells (10^13^) [2],[3]. It has been stated that the key to understanding the human condition lies in understanding the human genome [4],[5]. But given our intimate relationship with microbes [6], researching the human genome is now understood to be a necessary though insufficient condition: sequencing the genomes of our own microbes would be necessary too. Also, to better understand the role of microbes in the biosphere, it would be necessary to undertake a genomic study of them as well.

The study of microbial genomes started in the late 1970s, with the sequencing of the genomes of bacteriophages MS2 [7] and ϕ-X174 [8]. In 1995 microbiology took a major step with the sequencing of the first bacterial genome *Haemophilus influenza* [9]. The genomes of 916 bacterial, 1,987 viral, and 67 archaeal species are deposited in GenBank release 2.2.6. Having on hand such a large number of microbial genomes has changed the nature of microbiology and of microbial evolution studies. By providing the ability to examine the relationship of genome structure and function across many different species, these data have also opened up the fields of comparative genomics and of systems biology. Nevertheless, single organism genome studies have limits. First, technology limitations mean that an organism must first be clonally cultured to sequence its entire genome. However, only a small percentage of the microbes in nature can be cultured, which means that extant genomic data are highly biased and do not represent a true picture of the genomes of microbial species [10]–[12]. Second, very rarely do microbes live in single species communities: species interact both with each other and with their habitats, which may also include host organisms. Therefore, a clonal culture also fails to represent the true state of affairs in nature with respect to organism interaction, and the resulting population genomic variance and biological functions.

New sequencing technologies and the drastic reduction in the cost of sequencing are helping us overcome these limits. We now have the ability to obtain genomic information directly from microbial communities in their natural habitats. Suddenly, instead of looking at a few species individually, we are able to study tens of thousands all together. Sequence data taken directly from the environment were dubbed the metagenome [13], and the study of sequence data directly from the environment—metagenomics [14].

However, environmental sequencing comes with its own information-restricting price tag. In single organism genomics practically all of the microbe's genome is sequenced, providing a complete picture of the genome. We know from which species the DNA or RNA originated. After assembly, the location of genes, operons, and transcriptional units can be computationally inferred. Control elements and other cues can be identified to infer transcriptional and translational units. Consequently, we achieve a nearly complete and well-ordered picture of all the genomic elements in the sequenced organism. We may not recognize all the elements for what they are, and some errors may creep in, but we can gauge the breadth of our knowledge and properly annotate those areas of the genome we manage to decipher.

In contrast, the sequences obtained from environmental genomic studies are fragmented. Each fragment was obviously sequenced from a specific species, but there can be many different species in a single sample, for most of which a full genome is not available. In many cases it is impossible to determine the true species of origin. The length of each fragment can be anywhere between 20 base pairs (bp) and 700 bp, depending on the sequencing method used. Short sequence reads that are dissociated from their original species can be assembled to lengths usually not exceeding 5,000 bp; consequently, the reconstruction of a whole genome is generally not possible. Even the reconstruction of an entire transcriptional unit can be problematic. In addition to being fragmented and incomplete, the volume of sequence data acquired by environmental sequencing is several orders of magnitude larger than that acquired in single organism genomics.

For these reasons, computational biologists have been developing new algorithms to analyze metagenomic data. These computational challenges are new and very exciting. We are entering an era akin to that of the first genomic revolution almost two decades ago. Whole organism genomics allows us to examine the evolution not only of single genes, but of whole transcriptional units, chromosomes, and cellular networks. But more recently, metagenomics gave us the ability to study, on the most fundamental genomic level, the relationship between microbes and the communities and habitats in which they live. How does the adaptation of microbes to different environments, including host animals and other microbes, manifest itself in their genomes?

For us humans, this question can strike very close to home, when those habitats are our own bodies and the microbes are associated with our own well-being and illnesses: almost every aspect of human life, as well as the life of every other living being on the planet, is affected by microbes. We now have the experimental technology to understand microbial communities and how they affect us, but the sheer volume and fragmentary nature of the data challenge computational biologists to distill all these data into useful information.

In this article we shall briefly outline some experimental, technological, and computational achievements and challenges associated with metagenomic data, from sequence generation and assembly through the various levels of metagenomic annotation. We will also discuss computational issues that are unique to environmental genomics, such as estimating the metagenome size and the handling of associated metadata. Finally, we will review some studies highlighting the advantages of metagenomic-based research, and some of the insights it has enabled.

## Sampling

### Sample Size and Number of Samples

The first step in a metagenomic study is to obtain the environmental sample. Samples should represent the population from which they are taken. The problem in microbial ecology is that we are unable to see the organisms we are trying to capture. How many samples are enough?

To estimate the fraction of species sequenced, rarefaction curves are typically used. A rarefaction curve plots the number of species as a function of the number of individuals sampled. The curve usually begins with a steep slope, which at some point begins to flatten as fewer species are being discovered per sample: the gentler the slope, the less contribution of the sampling to the total number of operational taxonomic units or OTUs. For microbial samples, different OTUs are typically characterized by 16S (prokaryotic) or 18S (eukaryotic) rDNA, and are also referred to as ribotypes. Classification is rarely done in the field, so some initial estimate of species diversity by a pilot study or previous studies is desirable to gauge the number of samples needed to get a comprehensive picture of the OTUs in the sampled habitat. More of this will be discussed in the “Species Diversity” section below.

### Filtering

When filtering an environmental sample, as with any kind of filtering, the goals are: (1) get as much as you can of what you want and (2) leave out as much as you can from what you do not want. So if we are interested in bacteria only, our goal would be to filter out the smaller viroid particles, and the usually larger protists. Of course, this process will leave in the lysogenic phages and prophages, which are integrated in bacterial genetic material, as well as mimivirus particles, which are as large as some bacteria. On the other side of the size scale, small protists and large bacteria may overlap in size, making a full size-based separation impossible. Also, filamentous forms of bacteria that grow in multicellular colonies may also be filtered out owing to colony size exceeding that of the filter's pores.

Computational filtering can be used after sequencing. Genomic material that is obviously within the clades of interest can be filtered in using similarity searches against annotated sequence databases. Care must be taken, though, with false negatives: relevant genomic material may be filtered out in this fashion simply because homologs have never been deposited in existing databases. Another option would be to search for obviously false-positive sequence motifs, e.g., eukaryote material when only prokaryote material is to be analyzed. This technique can also be used to detect sample contamination.

### Recording Metadata

Keeping strict and comprehensive records of metadata is as important as the sequence data. Metadata are the “data about the data”: where the samples were taken from, when, and under which conditions. In microbial ecology, this commonly refers to physical, chemical, and other environmental characteristics of the sample's location. For example, an ocean sample metadata will typically include sampling date and time, depth, salinity, light intensity, geographical coordinates, pH, soluble gases, etc. In clinical microbiology, metadata would refer to the pathology, medical history, and vital statistics of the patient as well as the exact location and tissue from which the sample was taken, the sampling conditions, and so on.

Many metagenomic studies are driven by discovery and data mining, rather than by hypothesis. These studies seek statistically significant correlations between the metagenomic data and the habitat-associated metadata, which may lead to biologically significant discoveries. There is therefore a need to provide metadata in a form that is standard, comprehensive, and amenable to computation. For example, semantic information should be provided, wherever possible, in ontological form. A description of the environmental context and the experimental methods used is vital to enable comparative studies. As we shall see, genes or even “gene-less” sequence signatures are linked to habitats rather than to species. Finally, sequencing technology is rapidly improving, and the adoption of new sequencing methods will require the adoption of descriptors of those methods such as sequence coverage, quality, assembly programs that were used, and so on.

The Genomic Standards Consortium (http://gensc.org/) is an international group working to standardize the description of genomes and metagenomes and the exchange of genomic data and metadata. In a recent publication, a standard for the Minimum Information of Genomic and Metagenomic (MIGS/MIMS) metadata was suggested for adoption [15], and an associated markup language, the Genomics Contextual Data Markup Language or GCDML is under active development [16]. It is the consortium's aim that the MIGS/MIMS shall be adopted by journals as a publication requirement when genomic or metagenomic data are being deposited, akin to standards such as MIAME for microarray data [17] or PDB/mmCIF for structural biology data [18].

## Sequencing

### First, Second, and Third Generation Sequencing

Until recently, prokaryotic genomes have been typically sequenced using Sanger shotgun [19],[20] sequencing. The first step is shearing the DNA content of a genomic clone into random fragments, hence the “shotgun.” The fragments are then cloned into plasmid vectors that are grown in monoclonal libraries to produce enough genomic material for sequencing. The DNA is then sequenced using dye-termination methods. Repetition of this process ensures that all parts of the studied genome are sequenced, several times over. Assembly software is then used to assemble the sequence fragments into the whole genome. Theoretically any genome shorter than 5 Mbp can be assembled this way, although regions with large repeats tend to frustrate assembly algorithms. Therefore, regions with large repeats are often not incorporated into the whole genomic picture, leaving some gaps. Another disadvantage of shotgun sequencing is the “cloning bias.” Some genes cannot be incorporated into the library vector, usually because of toxicity to the vector expressing them [21]. This inability to be incorporated is typically mitigated by using more than one organism for cloning, or by using sequencing techniques that do not require cloning (see below) in second generation sequencing.

**Figure 1:**
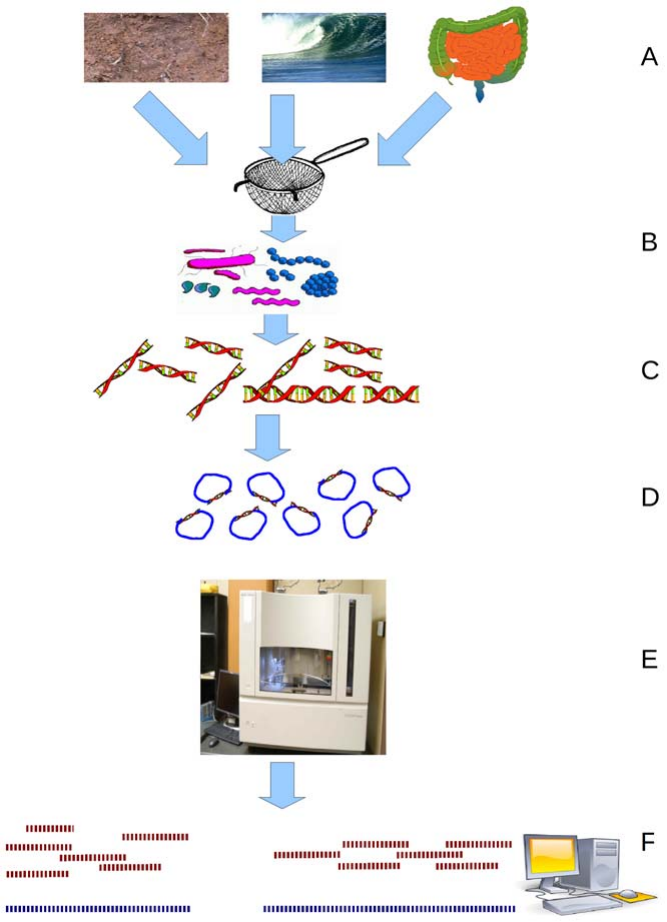
Environmental Shotgun Sequencing (ESS). (A) Sampling from habitat; (B) filtering particles, typically by size; (C) DNA extraction and lysis; (D) cloning and library; (E) sequence the clones; (F) sequence assembly.

In metagenomics, shotgun sequencing is done in the same manner as in clonal culture genomics. However, the raw genomic material does not come from a single organism: it comes from a community of microbes, hence the name environmental shotgun sequencing or ESS. Depending on our ability to sample, this DNA may provide only a partial genomic picture of the organisms in the environment, since the genomic material from the more abundant species dominates the sample. To obtain a better picture of the species composing the community, 16S rDNA or 18S rDNA for prokaryotic and eukaryotic samples, respectively, are sequenced separately using universal primers, see Figure 1. It should be noted that when using primers for rDNA to classify OTUs in an environmental sample, there are choices to be made regarding the primer sequence, especially when the studied OTU composition is expected to differ significantly from most known species, the so-called rare biosphere [22],[23]. In this case, there is the possibility that the primers used will be too different from the rDNA in the sample, which would result in many OTUs not being identified [24],[25].

Second generation sequencing methods have been rapidly gaining ground and are replacing Sanger sequencing for small sized genomes and environmental genomics. A common denominator among second generation methods is the generation of “polymerase colonies” or polonies [26],[27]. Polonies are PCR amplicons derived from a single molecule of nucleic acid. Thousands to millions of polonies, each with an effective reaction size of 10^−9^ l to 10^−12^ l can be amplified simultaneously, generating templates for sequencing. Following that, enzymatic reactions can be performed in parallel to sequence the nucleic acid material in the polonies. Polony-based methods produce considerably more sequences than Sanger sequencing, but those sequences are much shorter. Furthermore, each polony-based method has its own anomalies that should be accounted for when processing the data. See Table 1 for a comparison between the yield, fragment length, and run times of the different sequencers.

**Table 1:** Comparison of different sequencing technologies, taken from [34].

Sequencer	ABI 3730	Roche 454	Solexa^a^	SOLiD (mp, frag)^b^	HeliScope^c^
**Read length**	600–900	400–500	75–100	50	25–35
**Run time**	6–10 h	10 h	2–10 d	(4–7 d,8–14 d)	h
**Yield (Mbp)**	0.01	1	2,300–3,500/d	(500, 1,000)	105–140/h
**Cloning bias**	Yes	No	No	No	No
**Mate pair information**	Yes	No	Yes	Yes	No

a Based on the GA IIx. See full specifications at: http://www.illumina.com/systems/genome_analyzer.ilmn.

b mp, mate pair; frag, fragment. See https://products.appliedbiosystems.com/ SOLiD 3 Plus System.

c See: http://www.helicosbio.com/Products/HelicosregGeneticAnalysisSystem/HeliScopetradeSequencer/tabid/87/Default.aspx.

**Figure 2:**
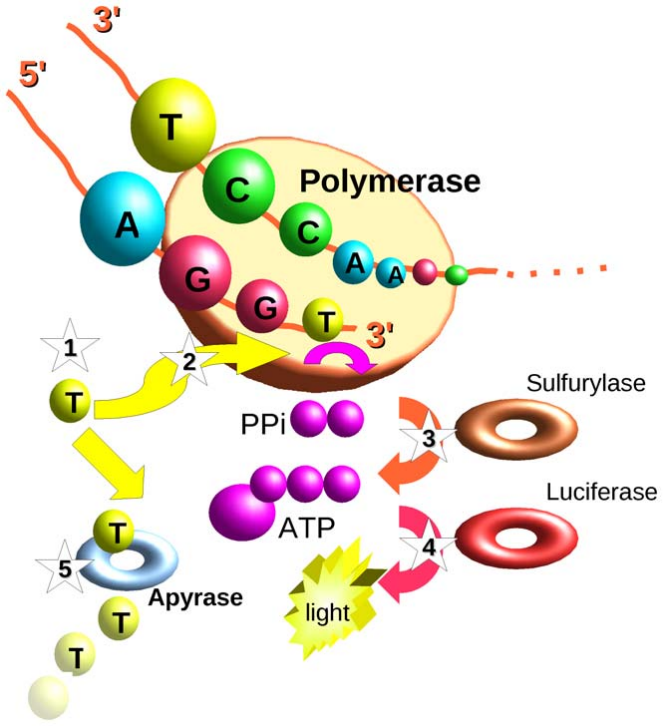
Pyrosequencing. Single stranded DNA template is first hybridized with the sequencing primer and mixed with the enzymes along with the two substrates adenosine 5′-phosphosulfate (APS) and luciferin. In each cycle, (1) one of the four nucleotides (dTTPi, in this case) is then added to the reaction. (2) If the nucleotide is complementary to the base in the template strand then the DNA polymerase incorporates it into the growing strand. (3) Pyrophosphate (PPi)—in an amount equal in molarity to that of the incorporated nucleotide—is released and converted to ATP by sulfurylase in the presence of APS. (4) ATP then serves as a substrate to luciferase, causing a light reaction. Photon emission is in equimolar quanta to the amount of nucleotide incorporated in a given cycle. (5) The excess nucleotides are degraded by apyrase.

In pyrosequencing (Figure 2) [28],[29], methods such as Roche 454 [30] sequencing is performed by polymerase extension of a primed template. Single nucleotide species are added at each cycle. If the particular nucleotide species added to the polymerase reaction pairs with the one on the template, the incorporation causes luciferase-based light reaction. The reaction chamber is then washed, and the cycle repeated. Several hundreds of thousands of wells containing material for sequencing are typically used in a single reaction. Second is the inability to read long mononucleotide repeats correctly.

ABI SOLiD and Illumina GAII sequencers produce even shorter reads: 25–100 bp, but very large volumes of DNA per sequencing run. As we shall see in the “Assembly” section below, despite the individual short read lengths, these technologies provide a viable alternative for sequencing whole genomes, by sheer volume of DNA sequenced. For further reading on second generation sequencing see [31]–[34].

Third generation sequencing, loosely defined as technology that is capable of sequencing long sequences without amplification, is in advanced development. There are encouraging signs that this technology might be available as early as 2011 [35]–[37].

## Assembly

When sequencing a whole genome, the reads are assembled into progressively longer contiguous sequences or contigs, and finally to the whole genome. Dealing with genomic data, we are used to analyzing long stretches of contiguous sequence data. This analysis lets us find not only open reading frames, but also operons, operational transcriptional units, their associated promoter elements, and transcription factor binding sites. Longer elements such as pathogenicity islands, and other mobile genetic elements are evident only when large fractions of the genome are assembled. The gain of information correlates with the length of the genomic elements. Table 2 shows the length of a genomic sequence, and the information that may be gleaned from it.

**Table 2:** The information contained in different lengths of genomic DNA.

Sequence Length (bp)	Genome Element
25–75	SNPs, short frameshift mutations
100–400	Short functional signatures
500–1,000	Whole domains, single domain genes
1,000–5,000	Short operons, multidomain genes
5,000–10,000	Longer operons, some *cis*-control elements
>100,000	Prophages, pathogenicity islands, various mobile insertion elements
>1,000,000	Whole prokaryotic chromosome organization

In contrast, in all but the most species-poor metagenome, a full assembly is not possible—first, because the sampling is incomplete, and many if not all species' genomes are partially sampled, if at all; second, because the species information itself is incomplete, and it is difficult to map individual reads to their species of origin. Therefore, the analysis of genomic elements using metagenomic data is generally limited to the first three or four rows in Table 2.

In this section, we will discuss assembly of metagenomic data, how information is extracted from partial assemblies, and how the extent of information gained can be estimated.

### Metagenomic Sample Coverage

#### Coverage

Coverage of a genome is defined as the mean number of times a nucleotide is being sequenced. Thus, 5× coverage means that each nucleotide in the genome is sequenced a mean number of five times. If we could sequence a genome in a single read, then 1× coverage would suffice for sequencing. Shorter read lengths (25–700, depending on sequencing technologies, see Table 1), necessitate more coverage, to ensure all reads overlap, and that those overlaps are unique enough to reconstruct the genome by assembling the fragments. If we treat DNA shearing and sequencing as random events, and our ability to detect and overlap between two truly overlapping reads does not vary between clones (when those are used), then we can use a Poisson distribution model to estimate the number of reads required to sequence an entire genome. This model is given by the Lander-Waterman equation [38]:
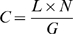



Where *L* is the read length, *N* is the number of reads, *G* is the genome length, and *C* is coverage as described above. The fraction of sequence covered would be given as:
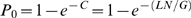



To get the number of reads sequencing fraction *P*_0_ of the genome
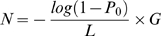



In an environmental sample containing *l* species, the metagenome size *G*_m_ is:
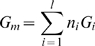



Where *G*_i_ is the size of any given genome in a sample containing *l* genomes, and *n*_i_ the number of copies of genome *g*_i_.

However the species that constitute the sample appear in different frequencies in the metagenome. Therefore a metagenome of size *G*_m_ composed of genomes of sizes *G*_1_ through *G*_k_ can be viewed as a sum of fractions. Each component genome of size *G*_i_ constitutes a fraction of *G*_m_:
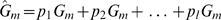
and:
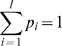



Where *p*_i_ is the fraction of copies of the genome of species *i* in the sample and *G*_i_ is the size of the genome of species *i*.

Using species-specific gene markers, usually small ribosomal subunit rDNA, it is possible to estimate the species diversity in the sample, and provide an estimate of the different *p*_i_ values. Nevertheless, full or sometimes even adequate coverage (as judged by the rarefaction curve) of a species-rich environmental sample may be unattainable, especially for the genomes of the less represented species [39]–[42]. We expand upon this subject in the “Species Diversity” section.

Jeroen Raes and his colleagues have suggested an effective genome size or EGS measure that includes multiple plasmid copies, inserted sequences, and associated phages and viruses [43]. EGS uses the density (counts per megabase) of single copy marker genes to extrapolate the EGS.
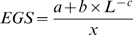



Where *L* is the read length, *x* is the marker gene density, *a*, *b*, and *c* are empirical parameters empirically derived from 154 simulated metagenomes and found to be 21.2, 4,230, and 0.733, respectively. Raes and colleagues derived this formula from several different metagenomes, providing a useful measure of central tendency for genome size using a metagenomic sample. Note that *a*, *b*, and *c* were derived from simulated metagenomes, Therefore, care must be taken in using the EGS formula above, since the parameters given only provide a snapshot of a particular simulation. It is probably better to use EGS as a framework, in conjunction with a metagenomic simulator such as MetaSim [44] to generate parameters more compatible with population estimates in one's own research. MetaSim enables the creation of a simulated genome from regular genomic files; this makes it useful for testing and assessing the performance of other programs that manipulate and analyze metagenomic data, such as assembly or annotation programs.

### Metagenome Assembly

**Figure 3:**
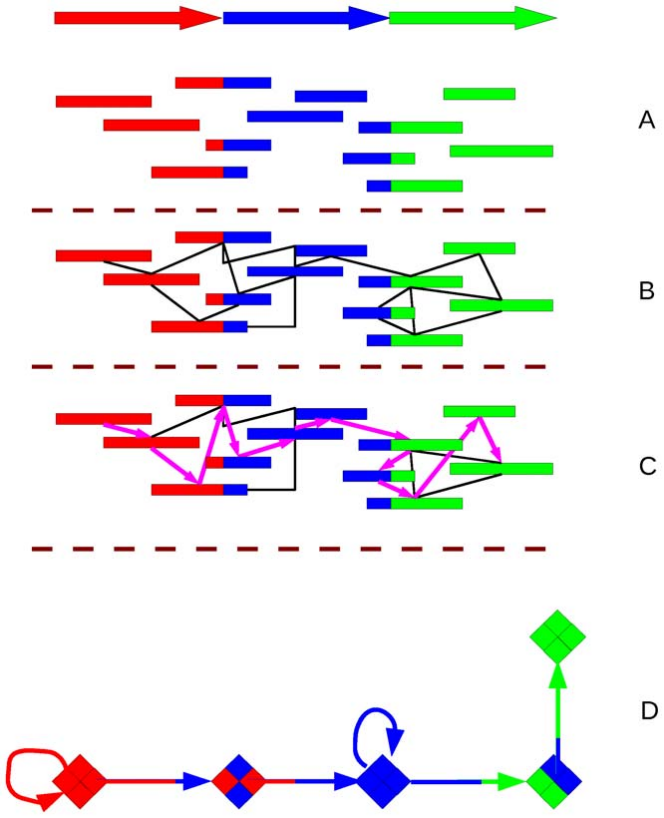
Fragment assembly. (A–C) Hamiltonian. (A) A sequence with overlapping reads; (B) Each read is represented as a vertex, with edges connecting the overlapping vertices; (C) the assembly solution is a Hamiltonian path (all vertices are visited, no vertex is visited more than once) through the resulting graph; (D) For short reads assembly, each vertex is a *k*-mer (or a hashed collection of *k*-mers), and the reads are threaded between vertices as edges. The solution is a Eulerian path, where each edge is visited once. Repeats are merged into a single edge. For detailed algorithms see [49], [50], [53]–[55].

In a genome project of a single organism or clone we can be certain that all extracted DNA fragments belong to the same genome, barring contaminants and extrachromosomal DNA. That is not the case when a metagenome is concerned. As we have just seen, coverage is usually incomplete, since environmental sequence sampling rarely produces all the sequences required for assembly. Furthermore, there is also the danger of assembling sequences from different OTUs, creating interspecies chimeras. Phrap, Forge, Arachne [45], JAZZ [46], and the Celera Assembler [47] are all assembly programs that were developed for single genome assembly from Sanger sequencing. They seem to provide good results even when assembling metagenomic sequence data from Sanger sequencing [48]. Most of these algorithms use mate-pair information for the assemblies. This information is used in assembly to check the scaffolds or the assembled intermediaries between raw reads and whole chromosomes. These assembly algorithms represent each read as a vertex and each detected overlap as an edge between the overlapping vertices. Finding the correct assembly is cast as a Hamiltonian path finding problem, for finding a path in a graph where each vertex is visited once (see Figure 3A–3C).

For short reads, however, this technique is not suitable. To establish adequate coverage, short reads need to be produced in large quantities, and their short lengths means that there are many identical, or nearly identical, reads. The plethora of reads makes representing the vertices as single reads impossible. Another problem is that the sheer volume of reads makes the graph large and unmanageable. The solution to a Hamiltonian path is an NP-complete problem, meaning that the time necessary for a solution grows exponentially with the number of nodes. So while it is possible to solve for a relatively low number of reads as are produced using Sanger sequencing, the problem becomes intractable with the large amounts of sequence data from second generation sequencers.

One solution is for the vertices to represent *k*-mer words with the reads themselves being the edges connecting the vertices. Since the vertices represent *k*-mers rather than reads, the high number of reads and their redundancy does not affect the number of nodes. Repeats exist in the graph only once, with links to the different start and end points. Searches for overlaps are simplified, as overlapping reads are mapped onto the same edge and can easily be followed simultaneously. Finally, since the reads are represented as edges rather than vertices, the solution is a Eulerian path, where each edge is visited once. Unlike a Hamiltonian path, a linear-time algorithm to solve a Eulerian path does exist, making the assembly problem tractable for large number of reads.

The EULER assembler [49],[50] was the first to present this technique using de Bruijn graphs. De Bruijn graphs are *n*-dimensional graphs of *m* symbols. For metagenomic assembly, *m* = 4 (A,T,G,C) and 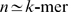 length. Theoretically, there are *m* ^n^ vertices, but the dimensionality can be greatly reduced by hashing the reads in the dataset to be assembled (see Figure 3). Other variations have since been published, adapting to short (100–200) [51],[52] and very short read lengths [53]–[55]. EULER and VELVET are available for download. Recently, Ye and Tang developed an assembly method that finds putative open reading frame (ORF) regions first, and then assembles those regions. This method, dubbed ORFome assembly, increases assembly accuracy for ORF regions at the expense of losing noncoding regions. Nevertheless, for many practical purposes this method is very useful, because it appears to have a better recovery rate, for coding regions only, than regular, whole genome assemblers [56]. For recent reviews on computational assembly methods see [57],[58].

## Gene Calling

Genes are the basic functional unit in the genome, which may constitute larger functional units such as operons, transcriptional units, and functional networks. Again, the incomplete and fragmentary nature of metagenomic data presents challenges to identifying genes. With Sanger random shotgun sequencing, whole genomes are rarely assembled, and in species-rich environments, many reads remain as singletons rather than being joined in contigs. In the Global Ocean Sampling (GOS) data, which were Sanger-sequenced, the mean number of whole reading frames per assembly is 4.7 [59].

Gene finding algorithms are trained to find whole ORFs and take into account information gleaned from large genomic stretches. For metagenomic data, however, this information is unavailable. Despite such drawbacks, Mavromatis and colleagues have shown that for a high complexity metagenomic dataset, gene prediction on assemblies can be as accurate as 85% of the originally predicted genes in the constituting genomes. For a low complexity set this goes up to 90% [48].

For genes with known homologs, BLASTing (using the Basic Local Alignment Search Tool) [60],[61] against known databases is a common approach. This approach informs of the existence of gene family members within a metagenome. BLAST cannot be used to find new families and new genes that have no homologs in known databases. For that, ab initio gene prediction tools are used. Those tools are mostly based on supervised learning and statistical pattern recognition methods. Most models use Markov models or Hidden markov models. Genemark.hmm is a program that uses inhomogeneous Markov models based on monocodon frequency analysis for gene calling [62]. When applied to metagenomic data, however, those methods lose sensitivity, because they often fail to identify partial ORFs that may be part of true genes. This is especially true when conventional gene calling methods are applied to raw Sanger fragments rather than to assemblies. Unsupervised methods are therefore required.

Yooseph and colleagues [59],[63] have used a different approach to gene finding when analyzing the global ocean survey data. They began with simple ORF identification of consecutive translatable regions that translate to at least 60 amino acids (aa). They then clustered those sequences using an all-against-all BLAST search, identifying clusters containing nonredundant sequences. In the next step, shadow ORFs were eliminated. Shadow ORFs are false ORFs in a different reading frame than the true ORF, but they overlap the true ORF and hence may be mistaken for a coding region. Yooseph and colleagues handled this by clustering all ORF candidates in the same reading frame and selecting the larger cluster as the one containing true ORFs, discarding the other ones as shadow ORFs. Finally, they removed ORF families with a KaKs *K*_a_/*K*_s_ ratio that is close to 1. The rationale for this step is that putative proteins that are seemingly under no selective pressure (positive or negative) are probably falsely identified. Gene families coding for proteins under selective pressure are expected to have a 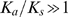 or 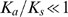.

It has been argued that one drawback of the incremental clustering method is that it increases specificity at the expense of sensitivity; that is, it may have an excess of false negatives due to the removal of putative ORFs that do not cluster well or do not cluster at all in the database [64]. As of today, however, there has not been a thorough comparative evaluation of gene calling methods on first or second generation sequence data.

## Species Diversity

### Measuring Diversity

**Figure 4:**
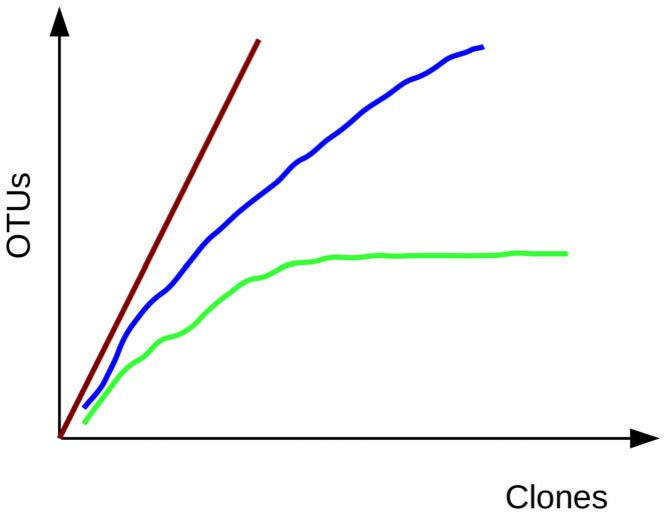
Rarefaction curves. Green, most or all species have been sampled; blue, this habitat has not been exhaustively sampled; red, species rich habitat, only a small fraction has been sampled.

In the “Sample Size” we discussed using 16S/18S rDNA for phylotyping and assessing species coverage using a rarefaction curve. Microbial ecology has many tools for assessing species diversity. Rarefaction curves are used to estimate the coverage obtained from sampling, see Figure 4. α-diversity, β-diversity, and γ-diversity are all well-established diversity indices used in ecology, including microbial ecology. α-diversity is the biodiversity in a defined habitat or ecosystem; β-diversity compares species diversity between habitats; γ-diversity is the total biodiversity over a large region containing several ecosystems. Here we will discuss the application of these indices to metagenomic data.

One way to calculate α-diversity is by using Shannon's index:
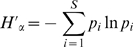
where:
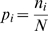



Where *S* is the total number of OTUs, *n*_i_ is the number of clones in each OUT, and *N* is the total number of individuals. *p*_i_ is the relative abundance of each OTU. 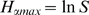.

#### Using different sequence markers for OTU identification

It should be noted that using 16S/18S rDNA as a proxy for OTU identification and counting is not without problems. First, rDNA has been criticized as an OTU marker, and evidence of horizontal gene transfer involving rDNA may confound its reliability even more [65]. Second, 16S rDNA may exist in multiple different sequence copies in a single bacterium: this would cause a variance in both the estimated individual bacterial count, and OTU numbers. It is commonly accepted that the mean number of bacterial ribosomal operons per genome is 4.1 [66], but in a recent publication it has been shown that 16S rDNA gene copy numbers may vary between 1 and 15 [67],[68]. Alternative markers, such as single copy housekeeping genes have been suggested as alternative or complementary species and population tally markers for bacterial genomes. The *rpoB* gene is a strong candidate [69], but *amoA*, *pmoA*, *nirS*, *nirK*, *nosZ*, and *pufM* have also been suggested in different contexts [67],[70]. The housekeeping functionality of these genes makes them less susceptible to horizontal gene transfer. However, these studies have shown that on a finer level the use of housekeeping genes does improve upon 16S rDNA alone, the use of 16S rDNA as a marker for OTU identification and count is still sufficiently accurate for many purposes. The use of housekeeping genes for OTU classification is primarily for those cases when 16S rDNA provides a lower resolution than when a high diversity of species is expected. Another case where a housekeeping gene is preferable to 16S rDNA is when the variation in the housekeeping gene matches the acceptable taxonomy better than the variation in the rDNA sequences. The use of non-rDNA phylogenetic markers has been applied to metagenomic data, showing that certain microbial communities evolve faster than others [71].

Epidemiologists classify bacterial serovars for pathogen verification using Multilocus Sequence Typing (MLST) [72],[73]. MLST is a technique by which several standardized housekeeping genes are selected for OTU typification. There is an online resource for MLST, including a database for OTU identification (http://www.mlst.net/). MLST has been used successfully in some metagenomic studies [74]. However, MLST appears to be more useful for a finer level substrain typification, rather than OTUs.

In the same vein, 18S rDNA can have different count numbers in microeukaryotes, with an even larger copy number variation between species than 16S rDNA counts in prokaryotes. Care must be taken to account for this copy number variation when assessing the cell count in eukaryotic samples [75],[76].

There are several software packages we found very useful for biodiversity analysis. The first is a general purpose population analysis software, EstimateS (8.0) [77]. EstimateS contains a rich set of biodiversity analysis modules, but for microbial analysis it requires preprocessing of sequence data to transform it into generic population data. MOTHUR [78] is tailored towards microbial diversity analysis and provides tools for transforming sequence data to population data. It is not as rich in functional modules as EstimateS, but for most diversity analyses (rarefaction curves, standard estimate indices) it is more than adequate. QIIME, an extension of PyCogent [79], is in beta, but testing by one of us (IF) has shown it to be a very powerful and versatile package for analysis of genomic and metagenomic microbial ecology data (http://qiime.sourceforge.net). A more specialized software geared to the analysis of viral metagenomic data is PHACCS [80].

### Binning

We wish to know not only who populates the sample, but also what the different OTUs are doing. We must therefore associate sequence data with the OTU of its origin. This analysis is called binning (placing the sequence in its correct “bin” or OTU). In many cases, suitable phylogenetic marker genes are missing either because rDNA sequences may be unsuitable (as in virus analyses), or may have been undersampled.

Here we will examine two binning strategies: composition-based binning and phylogenetic binning.

#### Composition-based binning

The GC content of bacterial genomes is being used routinely for higher-level systematics [81]. With the advent of ESS data, a finer resolution for classifying or binning sequences is called for. Markov models based on *k*-mer frequencies have shown to be quite powerful for statistical analyses of DNA sequences [82]. For example, tetranucleotides are being used by the TETRA [83] program in the following fashion. There are 4^4^ = 256 possible DNA tetranucleotides. For each tetranucleotide 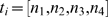, an expected frequency *E*(*t*_i_) can be calculated by means of a maximal-order Markov model:
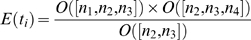



Where *O* is the observed count of the sub-trimers and dimer of the tetramer.

The level of over- and underrepresentation of each tetranucleotide is evaluated using *z*-scores:
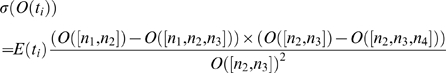


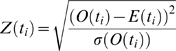



Where σ(*O*(*t*_i_)) is the variance in the tetranucleotide *t*_i_.

Composition-based binning is not error-free. The closer the OTUs in the studied metagenome and the more numerous they are, the higher is the frequency of misclassification errors. The strength of *k*-mer–based binning is that there are no reference sequences required for the actual binning: all the information is intrinsic. This makes *k*-mer a powerful tool for binning ORFan sequences: sequences that have few or no homologs and therefore no known function. Therefore, TETRA is independent of existing genomic data, since it does not require any training. PhyloPythia [84] is a supervised method that trains a set of support vector machines (SVMs) to bin sequences of a length greater than 1 kb, and thus not suitable for binning second generation sequences. It performs best when a training set is similar in phylotypic composition to the training set. Growing Self Organizing Maps or GSOM [85] and Seeded GSOM or S-GSOM [86] use a variant of the machine learning algorithm self-organizing maps. S-GSOM improves upon GSOM by extracting the flanking sequences of highly conserved 16S rDNA from the metagenome and using them as seeds to assign other reads on the basis of their compositional similarity. Both use frequencies of di- to penta-nucleotides for binning assignment.

Another composition-based method is codon-usage. An old technique in genomics, codon usage, can also be used for binning metagenomic data. Different species use different codon frequencies to encode the same amino acids, and this observation can be exploited to classify ORF sequences. Shani Tzahor and colleagues have developed a composite supervised method that uses both TETRA and codon usage statistics to classify fragments in the 100–300-bp range [87].

TETRA is available for download, and PhyloPyhtia is available as a Web site, with a downloadable version available by request. GSOM/S-GSOM does not seem to be available at this time.

#### Similarity-based binning

Another way to bin sequences is to find similarities to reference sequences that can be used to build a tree. This technique is useful when most sequences in the sample have significant similarities to reference sequences from known OTUs. Given an unannotated sequence *A*, and two annotated reference sequences *B* and *C*, and using the similarity function *sim*, let us consider the case where we have 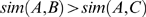; then, the sequence *A* will be placed on a node in the tree between *B* and *C*, and, in the case considered, closer to *B*. MEGAN [88] implements this method by reading a BLAST file output. Typically, the output is from the metagenomic reads or assemblies against nr, or any other sequence database that has a phylogenetic tree associated with it. MEGAN then assigns each read to the lowest common ancestor on the phylogenetic tree. This allows all sequences that have a homolog in nr to be assigned. Predicted gene sequences, having no homologs, are aggregated into their own single node on the tree. CARMA [89] is somewhat similar to MEGAN, but uses Pfam [90] as its source for taxonomic classification. It should be noted that a precise assignment to an OTU may not be possible in many cases. Nevertheless, unless it is an ORFan, the sequence can be placed in the species tree. The resulting picture of sequences on the species tree can provide an overview of the dominant species in the sample. Phymm [91] uses interpolated Markov models to characterize variable length DNA sequences by their phylogenetic grouping, unlike other methods. Phymm is trained on existing OTUs and learns which nucleotide length is best for classification. Also, Phymm does not leave reads unclassified, although that may impact its overall accuracy if there are many reads that cannot be accurately binned to any phylogenetic group.

As far as the usability of these software, CARMA will run on Unix-like environments, and its installation requires some third party software, and a rudimentary knowledge of Perl and MySQL. MEGAN runs in a Java virtual machine, and thus runs on almost out of the box Java-enabled platforms; it does require an installation of National Center for Biotechnology Information (NCBI)-formatted taxonomic reference database for lowest common ancestor mapping. Also, CARMA can run its own BLAST, whereas MEGAN requires a previously generated BLAST output as its input.

## Functional Annotation

Having assembled the metagenome and identified putative ORFs we would now like to understand the functional potential of the microbial community from where we derived the metagenome: what are these microbes capable of doing as a community? The first level of functional annotation is assigning biological functions to the ORFs. This task is highly challenging when applied to regular genomic data [92], and the challenge is compounded in metagenomic data where many ORFs are partial, and a large fraction have no annotated homologs. The second level would be discovering genes that constitute biological networks, such as metabolic pathways, in the data. The latter task is hampered by our inability to accurately associate each annotated ORF with a single species, which means it is sometimes hard to determine which component of a network comes from which organism. Nevertheless, binning can help to some extent. As we shall see in the “Case Studies” section below, several studies have been carried out and led to the successful discovery of complementary metabolic pathways from microbes that constitute a community.

In metagenomic samples the probability of not calling all genes is higher than in a fully assembled genome, since many ORFs may be partial, and thus invisible to regular gene calling software that require a full ORF. Therefore, one strategy for functional annotation would be to skip the gene calling step altogether. Instead, simply use six-frame translations on the reads provided. If the translations are reasonably long they may be ORFs. Even if they are short, but they are cut short because of being at the edge of a contig, they may still be partial ORFs. Now these putative partial ORFs can be searched for motifs, HMM profiles, and other sequence signatures that may indicate functionality. The rationale is that the probability of calling a false ORF that also includes a known sequence signature is negligible. Some metagenomic annotation programs use this rationale. For example, Motif EXtraction (MEX) [93] is an unsupervised motif creation method that is successful in identifying enzymes in genomic and metagenomic data [94],[95]. Short, enzyme-specific peptides are identified in an unsupervised learning stage. They are subsequently associated with certain functions, in the supervised learning stage. The reason an unsupervised stage takes place is because, in many cases, new motifs can be identified within ORFans, even though their functional association may be unknown.

Even unassembled single reads (singletons) may be used to infer functional information, being long enough to find short motifs or significant BLAST hits. BLASTing singletons and annotating the results without assembly or postassembly has its use. Two versatile and useful annotation pipelines for metagenomics that implement the annotation principles outlined above are MG-RAST [96] and RAMMCAP [97]. MG-RAST accepts a 454 dataset as input, normalizes it (removes artefactual duplicate sequences, a known problem with 454 sequencing), and then performs gene calling and annotation by a variety of sequence similarity searches (mainly BLAST) against various sequence databases, including 16S rDNA. It then produces statistics on species associations and on metabolic pathway associations using the SEED subsystems database as its guideline. RAMMCAP uses the fast clustering algorithm CD-HIT [98] to cluster translated ORFs by high sequence similarity. The rationale is that many similar putative ORFs strengthen the hypothesis that they are indeed real ORFs. Optionally, CD-HIT also serves to reduce the volume of data to be annotated by picking representatives from identical or nearly identical sequences and annotating only the representative sequences. The annotation is then transfered to the highly similar sequence in each similarity-based cluster. The sequences are then compared to the profile HMM databases Pfam [90] and TIGRfam [99] using HMMer (http://hmmer.janelia.org/) for functional annotation.

## Comparative Metagenomics

Comparing two or more metagenomes is necessary to understand how genomic differences affect, and are affected by, the abiotic environment. There are several sequence-based traits that can be compared: GC content was compared between marine and soil samples [59], microbial genome size [43], taxonomic [71], and functional content (e.g., [100]). Many comparative analyses, pairwise or multiple, make use of ordination statistics as when several metagenomic datasets are involved, or when several types of metadata are hypothesized to affect the observed compositions of the metagenomic populations. Principal component analysis (PCA) and nonmetric multidimensional scaling (NM-MDS) are typically used to visualize the data and to reveal which factors affect the observed data most (e.g., [101],[102]).

We mentioned MEGAN before as a binning software. MEGAN can also be used to compare the OTU composition of two or more frequency-normalized samples [103],[104]. MG-RAST provides a comparative functional and sequence-based analysis for uploaded samples, whereas IMG/M provides similar analysis for metagenomes that exist in the IMG/M site [105]. RAMMCAP also provides the ability to compare metagenomes. Other software used for the comparison of microbial populations based on phylogenetic data are UniFrac [106] and MetaStats [107], the latter being suitable for preprocessed clinical metagenomic data. Galaxy, an online workbench for the analysis of genomic data, can also perform some comparative metagenomic analysis, as well as taxonomic mapping [108]. ShotgunFunctionalizeR [109] is a stand-alone analysis tool for metagenomics samples written in R [110]. The megx.net resource includes include MetaMine [111] for annotating genes using neighboring ORF information, and MetaLook [112] for organization of sequences using customized habitat criteria. CAMERA (http://camera.calit2.net) offers to BLAST the user's sequences against 40 existing genomic and metagenomic datasets. CAMERA also serves as an archive for select metagenomic datasets generated by marine microbial research funded by the Gordon and Betty Moore Foundation. All of these sites appear to be in a state of flux, with promised new functionalities to be added soon and with datasets constantly being updated.

We mentioned the importance of standardized recording of metadata in the Recording Metadata section above. Comparative analysis is where the importance of metadata comes into play: in order to properly compare between different environments, we need a common vocabulary describing the abiotic components. To date we do not know of software that provides a comparison between metadata or a comparative correlation between metadata and sequence data, although several such comparisons have been performed (see “Case Studies” section below).

## Applications

In this section we will discuss a few studies involving metagenomics. We chose these studies because each one illustrates a different insight that is derived from using metagenomics.

### Correlations between Environmental Data and Metadata

The study of the effects of the environment on microbes is as old as microbiology itself. Antoni van Leeuwenhoek noted that the “animalcules” scraped from his mouth and that he viewed under his microscope were gone or were immobile after he drank hot coffee. Leeuwenhoek was the first to describe a correlation between temperature change and organism viability [113]. Ever since then, microbe species distribution, genetics, pathogenicity, virulence, colonization—indeed every aspect of microbial life—has been correlated with habitat traits such as temperature, salinity, pH, nutrient content, etc. Traits of host-borne microbes have been correlated with the host species, age, habitat, behavior, feeding habits, host organs chosen for settlement/pathogenicity, and, of course, clinical symptoms and many other traits.

With the advent of metagenomics, we are now able to study the genomic potential of a bacterial community and how it is affected by and affects its habitat. Many metagenomic studies have looked to some extent at correlations between sequence data, environment, and environmental attributes in an attempt to gain biological insight. One notable study by Turnbaugh and colleagues looked at the connection between the gut microbiome and obesity. The authors discovered that the metagenome in obese mice was enriched in carbohydrate active enzymes over that of lean mice. A separate biochemical experiment confirmed that the microbiome in obese mice has a larger energy harvesting capacity than in lean mice. They concluded that the gut microbiome contributes to obesity through this feed-forward cycle [100].

Studies such as those presented above looked at bivariate correlations: obesity and carbohydrate active enzyme enrichment. One recent study by Gianoulis and colleagues suggests how to locate multivariate correlations between metagenomic data and environmental attributes [114]. At the same time, environmental factors may combine in unexpected ways revealing new insights. Gianoulis and colleagues have identified covariation in amino acid transport and cofactor synthesis in nutrient-poor ocean areas, suggesting that limiting amounts of cofactor can (partially) explain increased import of amino acids in nutrient-limited conditions.

### Understanding Symbiosis

In many cases, symbiotic bacteria living in an animal host consist of a small number of species, which are often phylogenetically distant. Because they are few species and the phylogenetic distance makes their sequences relatively easy to bin, metagenomics is useful for studying symbionts. Eisen and his colleagues sequenced ESS data from bacterial symbionts living in the glassy-winged sharpshooter, which is an insect that lives solely on tree sap, a nutrient poor diet. By binning the ESS data they inferred that one symbiont synthesizes amino acids for the host insect, while another synthesizes cofactors and vitamins [115]. Not only that, but the symbiont providing the vitamins lacks some amino-acid synthetic pathways, and the symbiont providing the amino-acid synthetic lacks the ability to synthesize the vitamins. Thus, both symbionts complement each other's metabolic deficiencies, as well as feeding their host. Another study of the marine gutless worm *Olavius algarvensis* has revealed the different roles of its four symbionts in generating nutrients and processing the worm's waste [116]. None of the symbionts in the insect or in the worm study could be cultured under the reported conditions. Metagenomics thus became the chosen avenue for these studies.

### Enriching Gene Families

Another type of study enabled by metagenomics is the search for new members of a gene family. Metagenomics has opened up the floodgates of genomic material. Consequently the laborious hen-pecking for exemplars to enrich a studied gene family from known cultured species, has been replaced by the laborious computational filtering of appropriate exemplars from millions of environmental sequences. The previously small bacterial Eukaryotic Protein Kinase Like (ELK) family has been enriched several folds by the Global Ocean Sampling (GOS) project. Many new members of known families were identified, as well as new families. Within the protein sequences, four new residues of unknown function were found to be conserved, setting the stage for future functional studies of this family [117].

### Metagenomics and Environmental Virology

Outnumbering living microbes, viruses are the most abundant biological entity on Earth: there are an estimated 10^30^ tailed bacteriophages in the biosphere [118]. In marine environments, viruses constitute 94% of all nucleic-acid containing particles, although owing to their small size they are estimated to constitute only 5% of the biomass. Metagenomic studies have enriched our knowledge of viral diversity and the role viruses play as facilitators of microbial genetic diversity. Sequence similarity analyses of viral metagenomic data have shown that approximately 90% of the sequences have no similarity to GenBank sequences, telling of an underrepresentation of viral sequence data in sequence databases [119].

Transduction—the transfer of genetic material via a viral vector—is known to be a strong contributer to genetic diversity in prokaryotes. Metagenomic studies help us assess the magnitude of virally contributed genetic diversity. For example, the existence of photosynthetic genes in cyanophages—viruses infecting cyanobacteria—has been known for some time [120],[121]. However, metagenomic studies have revealed the extent of this phenomenon: it is estimated that 60% of the psbA genes, a component of Photosystem I, in surface water are of phage origin. Another metagenomic study revealed the existence of whole photosynthetic cassettes in cyanophages, which may increase host fitness by supplementing and enhancing existing cyanobacterial photosystems. The latter findings were enabled by the metagenomic data from Global Ocean Sampling (GOS). Surveying these data using simple sequence similarity analyses and chromosomal gene location have revealed the existence of Photosystem I genes in cyanophages, and the extent of their distribution [94],[122].

Clinical virology also stands to benefit from metagenomic analysis [123]. Indeed, recent molecular-based discoveries of highly prevalent viral infections caused by anellovirus [124] and GBV-C [125] highlight the need for a better understanding of the human viral flora.

The computational analysis of viral metagenomic data is particularly challenging. First, viruses may exist as a chromosomal insert, such as prophages, which are incorporated in the host genome. This incorporation confuses the ability to distinguish viral genomic elements from the host. Furthermore, when filtering exclusively for viral particles, prophage elements are lost. Second, viruses have no distinct phylogenetic marker gene, equivalent to the small ribosomal subunit rRNA in prokaryotes or eukaryotes. The lack of a consensual marker gene hampers phylogenetic and diversity analysis. Third, as stated above, most viral genes have no annotated homolog in sequence databases, which impedes functional analysis and indeed the identification of viral genes for what they are. Indeed, by some estimates the majority of ORFans in the biosphere is due to lateral gene transfer of viral origin [126] and the fact that phage-induced lateral gene transfer contributes in a major way to microbial diversity [127].

## The Future

We are in the midst of the fastest growing revolution in molecular biology, perhaps in all of life science, and it only seems to be accelerating. Sanger sequencing has been with us for over three decades. High-throughput 3730 sequencing has been around for 8 years, Roche 454 instrumentation has been available for 6 years, and Illumina GA for 3 years. The latter two methods have enabled us to generate more sequence data than Sanger sequencing has. We are still coming to grips with the large volume of data, and how to analyze it. Assembly, quality control, binning, and annotation all require ingenious algorithms combined with the latest computational power. It appears that sequencing technology is changing almost faster than the associated computational techniques can keep up. There are many indications that within a few years, short-read second generation sequencing may be outdated. Third generation sequencing that will enable the sequencing of a single chromosome in a single pass with few or no fragments should be established very soon [35],[36]. Does this plausible obsolescence of second generation sequencing change current metagenomic computational challenges? For some applications, assembly algorithms may be less warranted, but for species-rich samples, we may not be able to rely solely on third-generation sequencing for good sampling. Coverage assessment, gene finding, binning, and annotation will still be necessary.

The BASE technology from Oxford Nanopore is able to differentiate between cytosine and methyl-cytosine during sequencing [37]. Methylation acts as a primitive immune system in bacteria [128], and as an expression control mechanism in eukarya [129]. This additional epigenetic information has been mostly unavailable in sequencing projects due to an inability to obtain it in a high-throughput fashion. Pyrosequencing already offers a capability for quantitative methylation [130] and in all likelihood methylation data will be soon made available routinely along with the four base data, and the associated bioinformatics would need to address that.

Another growing problem is that of data management. Sequencing centers are working to equip themselves with computational infrastructure to meet the flow of sequence data. However, many research institutes who request the sequencing do not have the computational infrastructure needed to deal with analysis and long-term storage of these data. The sheer volume of data raises new constraints on its transfer and analysis. These challenges would have to be met by concerted efforts of life scientists, computer scientists, engineers, and funding agencies [131],[132].

Genomic data tell us what an organism is capable of doing, i.e., its genomic potential. What it is actually doing at a given time-frame is discovered by examining transcription (mRNA) and translation (protein) data. In the world of microbial communities, those studies have been dubbed metatranscriptomics and metaproteomics, respectively. These two fields are outside the scope of this review, but note that they too are very much in a development boom, technologically and computationally [133]–[135].

We hope this primer has been useful and informative. Because computational metagenomics is changing rapidly, we call upon the readers of this article who are knowledgeable in the subject to use the comment section of *PLoS Computational Biology* to provide updated information.

**Box 1. Glossary of terms**

**Binning** Clustering sequences based on their nucleotide composition or similarity to a reference database

**Contig** A set of overlapping DNA segments

**Coverage (in sequencing)** The mean number of times a nucleotide is sequenced in a genome

**ESS** Environmental Shotgun Sequencing

***K*** **_a_/** ***K*** **_s_** The ratio of the rate of nonsynonymous substitutions (*K*_a_) to the rate of synonymous substitutions (*K*_s_), which can be used as an indicator of selective pressure acting on a protein-coding gene

**Mate pairs** Sequences known to be in the 3′ and 5′ of a contig from a single clone

**Metadata** Definitional data that provide information about or documentation of other data

**Metagenome** The DNA obtained from uncultured microorganisms

**Metagenomics** The study of genomic DNA obtained from uncultured microorganisms

**Metaproteomics** The study of protein molecular data obtained from environmental samples using proteomics techniques

**Metatranscriptomics** The study of transcription sequence data obtained from environmental samples

**ORFan** An ORF that has no (or few, depending on definition) homologs in other organisms

**OTU** Operational taxonomic unit, species distinction in microbiology. Typically using rDNA and a percent similarity threshold for classifying microbes within the same, or different, OTUs

**Ontology** A formal representation of a set of concepts and the relationships between them. Ontologies are used to create a consensual unambiguous controlled vocabulary

**Polony** Discrete clonal amplifications of a single DNA molecule, grown in a gel matrix. The clusters can then be individually sequenced, producing short reads. Polony-based sequencing is the basis of most second generation sequencers

**Rarefaction curve** A curve describing the growth of a number of species discovered as a function of individuals sampled

**Ribotype** A phylotypic classification based on rDNA sequences

**Scaffold** A series of contigs that are in the right order but not necessarily connected in one contiguous stretch

**Shadow ORF** An incorrectly identified ORF that overlaps the coding region of the true ORF
